# Pharmacological Targeting of the ER-Resident Chaperones GRP94 or Cyclophilin B Induces Secretion of IL-22 Binding Protein Isoform-1 (IL-22BPi1)

**DOI:** 10.3390/ijms20102440

**Published:** 2019-05-17

**Authors:** Paloma Gómez-Fernández, Andoni Urtasun, Ianire Astobiza, Jorge Mena, Iraide Alloza, Koen Vandenbroeck

**Affiliations:** 1 Neurogenomiks Group, Department of Neuroscience, University of the Basque Country (UPV/EHU), 48490 Leioa, Spain; paloma.gomez.fernandez@gmail.com (P.G.-F.); a.urtasun.arricaberri@gmail.com (A.U.); nireaspe@hotmail.com (I.A.); jorgemena65@outlook.com (J.M.); iraide.alloza@ehu.eus (I.A.); 2 IKERBASQUE, Basque Foundation for Science, 48013 Bilbao, Spain

**Keywords:** IL-22BP, isoform, IL22RA2, ER, UPR, GRP94, gp96, GRP78, BiP, cyclophilin, geldanamycin, cyclosporin A

## Abstract

Of the three interleukin-22 binding protein (IL-22BP) isoforms produced by the human *IL22RA2* gene, IL-22BPi2 and IL-22BPi3 are capable of neutralizing IL-22. The longest isoform, IL-22BPi1, does not bind IL-22, is poorly secreted, and its retention within the endoplasmic reticulum (ER) is associated with induction of an unfolded protein response (UPR). Therapeutic modulation of IL-22BPi2 and IL-22BPi3 production may be beneficial in IL-22-dependent disorders. Recently, we identified the ER chaperones GRP94 and cyclophilin B in the interactomes of both IL-22BPi1 and IL-22BPi2. In this study, we investigated whether secretion of the IL-22BP isoforms could be modulated by pharmacological targeting of GRP94 and cyclophilin B, either by means of geldanamycin, that binds to the ADP/ATP pocket shared by HSP90 paralogs, or by cyclosporin A, which causes depletion of ER cyclophilin B levels through secretion. We found that geldanamycin and its analogs did not influence secretion of IL-22BPi2 or IL-22BPi3, but significantly enhanced intracellular and secreted levels of IL-22BPi1. The secreted protein was heterogeneously glycosylated, with both high-mannose and complex-type glycoforms present. In addition, cyclosporine A augmented the secretion of IL-22BPi1 and reduced that of IL-22BPi2 and IL-22BPi3. Our data indicate that the ATPase activity of GRP94 and cyclophilin B are instrumental in ER sequestration and degradation of IL-22BPi1, and that blocking these factors mobilizes IL-22BPi1 toward the secretory route.

## 1. Introduction

Interleukin-22 (IL-22) is a critical cytokine for host immunity and barrier homeostasis during infection and inflammatory diseases. It induces proliferative and anti-apoptotic signaling pathways as well as antimicrobial molecules, enhancing tissue regeneration and host defense [1]. A dual nature—exhibiting both pro- and anti-inflammatory effects—has been assigned to it [2,3]; moreover, IL-22 has been identified as a cancer-promoting cytokine [4,5]. It is mainly produced by cells from the innate and adaptive immune systems; however, the receptor through which it signals is limited to non-hematopoietic cells, mainly cells of epithelial origin cells [6]. Apart from the membrane receptor, IL-22 also binds to interleukin-22 binding protein (IL-22BP), a soluble decoy receptor capable of blocking IL-22 biological activity [7,8,9,10,11]. The human *IL22RA2* gene that codes for this soluble receptor co-expresses three transcript variants through alternative splicing (*IL22RA2v1*, *IL22RA2v2*, and *IL22RA2v3*) that encode distinct isoforms (IL-22BPi1, IL-22BPi2, and IL-22BPi3, respectively) [7,8,9]. By contrast, the rat and mouse genes only express a single variant, which is the homolog to the human *IL22RA2v2* [10,11]. Among the three isoforms produced in humans, the literature confirms isoform 2 (IL-22BPi2) as the main product and the one that exhibits highest affinity for IL-22, compared to the membrane-bound receptor IL-22R [12,13,14]. In addition, a shorter isoform, IL-22BPi3, is also capable of neutralizing IL-22 activity with lower affinity than IL-22BPi2, but higher affinity than that of the IL-22R [12,15]. Recently, we found that the longest isoform, IL-22BPi1, is not capable of interacting with IL-22, and is not efficiently secreted and largely retained in the endoplasmic reticulum (ER). IL-22BPi1 displayed hallmarks of a misfolded protein and induced the unfolded protein response (UPR) [16].

Similar to IL-22, both inflammatory and protective functions have been attributed to IL-22BP. Targeting the IL-22/IL-22BP axis is emerging as an attractive approach to prevent pathology associated with conditions in which IL-22 is known to be driving disease progress. Neutralizing IL-22 antibodies or recombinant IL-22BP are under investigation as promising therapeutic tools (https://clinicaltrials.gov/ct2/home). Specifically, inhibition of IL-22BPi2 and IL-22BPi3 in inflammatory bowel diseases may be useful for enhancing the suboptimal protective actions of IL-22 [17]. Given that *Il22ra2*-deficient mice experience a more benign course of disease in the experimental autoimmune encephalomyelitis (EAE) model, Laaksonen and colleagues [18] suggested that inhibition of IL-22BPi2 is worthy of exploration in neuroinflammation and multiple sclerosis. On the other hand, enhancement of IL-22BPi2 and IL-22BPi3 production may be indicated to control the deleterious actions of IL-22 in psoriasis [19] and intestinal tumorigenesis, where IL-22BP prevents long-lasting proliferative actions of IL-22 on malignant epithelial cells [20]. 

The possibility to modulate IL-22BP isoforms levels by directly interfering with their folding or secretion has, so far, not been studied. The ER machinery has been widely scrutinized as a therapeutic target for the treatment of diseases in which dysregulation of the UPR is involved, including neurodegenerative, autoimmune, cardiovascular diseases, as well as for the prevention of acute rejection and cancer treatment [21,22,23]. Secretion of both IL-22BPi1 and IL-22BPi2, but not IL-22BPi3, is dependent on the functionally intact luminal ER chaperones GRP78 and GRP94 [16]. Cyclophilin B, an ER peptidylprolyl isomerase, was also detected in the IL-22BPi1 and IL-22BPi2 interactomes by immunoblot [16]. Could interference with these ER factors modulate secretion of IL-22BPi2, the main IL-22 antagonist isoform? Disruption of GRP78 may be off limits due to its central role in UPR and the dependency of numerous secretory and membrane proteins on its activity [24]. However, GRP94 has a more selective client base of proteins, and the prospect of selectively blocking its activity in protein folding by pharmacological agents constitutes an area of active investigation [24,25]. While paralog-specific inhibitors of HSP90-type chaperones are in development [26,27], geldanamycin, a 1,4-benzoquinone ansamycin, is known to inhibit various homologs of HSP90, including GRP94, by binding to the characteristic ADP/ATP pocket, and is therefore useful for testing proof-of-concept [24,28]. Cyclophilin B displays an ability to bind the immunosuppressive drug cyclosporine A (CsA) [29]. CsA induces secretion of cyclophilin B into the culture medium, but not that of other ER chaperones, which de facto depletes the former’s ER levels [30] and facilitates scrutiny of its net effect on IL-22BP secretion. 

In this study, we investigated the effect of GRP94 and cyclophilin B targeting drugs on secretion of IL-22BP isoforms. 

## 2. Results

### 2.1. GRP94 and Cyclophilin B Are Co-Expressed with IL22RA2 in Monocyte-Derived Dendritic cells

**Figure 1:**
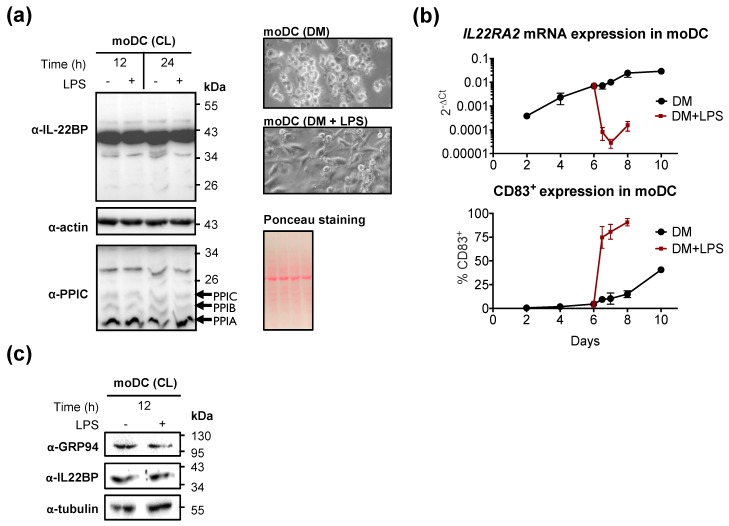
Detection of cyclophilin B and GRP94 in monocyte-derived dendritic cells (moDCs). (**a**) CD14^+^ monocytes were isolated from peripheral blood mononuclear cells (PBMCs) and differentiated into immature moDCs for 6 days. Cells were harvested at the indicated times following cultivation in differentiation medium (DM) supplemented (or not) with lipopolysaccharide (LPS) on day 6, and immunoblotted for detection of IL-22BP (anti-IL-22BP antibody), and cyclophilins A, B, and C, respectively indicated as PPIA, PPIB, and PPIC (the anti-PPIC antibody used detects the endoplasmic reticulum (ER) cyclophilins B and C, as well as the cytosolic cyclophilin A [34]) using actin and Ponceau staining as loading controls. (**b**) *IL22RA2* mRNA expression and maturation surface marker (CD83) were measured by qPCR and flow cytometry, respectively (mean ± SEM, *n* = 2). The morphology of moDCs stimulated with LPS for 12 h showed elongated cell bodies and increased adherence compared to non-stimulated moDCs; cells were photographed using a digital camera assembled on a bright-field inverted microscope. Original magnification was 40×. (**c**) Detection of GRP94 and IL-22BP by immunoblot in moDCs matured, or not, for 12 h on day 6 with LPS. Tubulin was used as loading control.

*IL22RA2* mRNA is upregulated in immature monocyte-derived dendritic cells (moDCs) and downregulated following maturation [15,16,20,31,32,33]. Expression of cyclophilin C which, like cyclophilin B, is a luminal ER-resident protein [34], is upregulated during the differentiation of CD14^+^ monocytes to moDCs [35]. We analyzed, by Western blot, the expression of IL-22BP and cyclophilins B and C, in immature and lipopolysaccharide (LPS)-matured moDCs (Figure 1a). Maturation of moDCs was verified by increased expression of CD83 [36] (Figure 1b) and changes in cell morphology (Figure 1a, photo insets). While maturation of moDCs with LPS strongly suppressed *IL22RA2* mRNA (Figure 1b), a ~40 kDa anti-IL-22BP immunoreactive band, as well as bands representing cyclophilin B and C remained constant (Figure 1a). Similar observations were made for GRP94, that is expressed at similar levels in both immature and mature moDCs (Figure 1c). Thus, cyclophilin B and GRP94, and their targets IL-22BPi1 and IL-22BPi2 [16], are co-expressed in moDCs (Figure 1a,c). 

### 2.2. GRP94 Inhibitors Enhance IL-22BP1 Secretion

**Figure 2:**
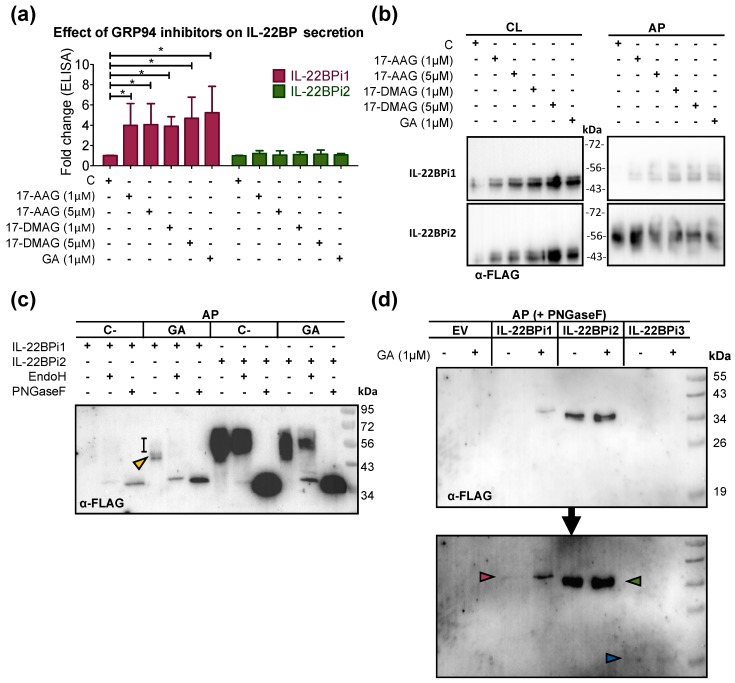
GRP94 inhibitors enhance IL-22BPi1 secretion. (**a**) HEK293 cells were transiently transfected with equal quantities of the indicated expression plasmids; 24 h later, cells were pretreated with the indicated concentrations of 17-dimethylaminoethylamino-17-demethoxygeldanamyin (17-DMAG), 17-allylamino-17-demethoxygeldanamycin (17-AAG), or geldanamycin (GA) for 4 h, or left untreated, and cell monolayers were then carefully washed five times with complete culture medium. Fresh complete culture medium was added, containing the same compounds at the same concentrations. IL-22BP protein levels were measured in the conditioned medium (CM) after 20 h, using ELISA (mean ± SEM; *n* = 5; * *p* < 0.05 by paired *t*-test). (**b**) Same as in (a), with the exception that CM was removed 16 h after transfection, cells were carefully washed five times with prewarmed serum-free medium (SFM) to remove abundant serum proteins, and fresh SFM containing L-glutamine was added for a further 4 h prior to acetone precipitation and recovery of cell lysates (CL). Acetone precipitates (APs) and CL were analyzed by immunoblot against FLAG Ab, which detects the C-terminal FLAG-tag on the IL-22BP isoforms. (**c**) HEK293 cells were transiently transfected with identical quantities of the indicated expression plasmids. Experimental procedures were as described in (b). AP was incubated with either PNGase F or Endo H, or left untreated. Proteins were immunoblotted and detected with anti-IL-22BP Ab. The orange arrowhead indicates the main Endo H-sensitive high-mannose-type glycoform of IL-22BPi1, while the Endo-H-resistant smear is indicated with a vertical black line. C-, negative control. (**d**) HEK293 cells were transiently transfected with identical quantities of the indicated IL-22BP expression plasmids. Experimental procedures as in (b). APs were treated with PNGase F. Proteins were immunoblotted and detected with FLAG Ab. Since IL-22BPi3 is difficult to detect, even in APs [16], membrane was overexposed for visualization. Red, green, and blue arrowheads indicate IL-22BPi1, IL-22BPi2, and IL-22BPi3, respectively.

We analyzed the effect of geldanamycin (GA) and its more stable or water-soluble analogs 17-allylamino-17-demethoxygeldanamycin (17-AAG) and 17-dimethylaminoethylamino-17-demethoxygeldanamycin (17-DMAG), respectively, on the secretion of IL-22BPi1 and IL-22BPi2 from transiently transfected HEK293 cells. As measured using ELISA, all three GA analogs significantly increased the secretion of IL-22BPi1 but not that of IL-22BPi2, and the inhibitory effect was maximal at drug concentrations of 1 μM (Figure 2a). As demonstrated before and confirmed here (Figure 2b), IL-22BPi1 is not detectable by Western blot in acetone precipitates (APs) of the medium of transfected cells [15,16]. Interestingly, GA and its analogs enhanced secretion of IL-22BPi1 to the point where it became visible in Western blots of APs as a series of 50 to 56 kDa bands (Figure 2b); also, intracellular levels of IL-22BPi1 were higher in the presence of GA analogs. No such effects were seen for IL-22BPi2. We asked whether the multiple secreted IL-22BPi1 bands corresponded to distinct glycoforms. Intracellular IL-22BPi1, similarly to IL-22BPi2 and IL-22BPi3, contains only high-mannose-type *N*-glycans [15,16]. In immunofluorescence microscopy of untreated cells, poorly secreted IL-22BPi1—in contrast to IL-22BPi2 and IL-22BPi3—does not colocalize with Golgi markers and appears confined to the ER [16]. However, the secreted form of IL-22BPi2 contains exclusively complex-type sugar chains, and this corroborates its secretory transit via the Golgi apparatus, which is known to contain the necessary glycosyltransferases for complex glycan attachment [16]. As shown in Figure 2c, IL-22BPi1 secreted in the presence of GA was sensitive to both Endo H (which cleaves only mannose-rich glycan chains) and PNGase F deglycosylation (which cleaves all high-mannose, hybrid, and complex glycan chains), and compatible with high-mannose-type *N*-glycans (Figure 2c). However, PNGase F treatment generated a more intense signal than Endo H, suggesting that part of IL-22BPi1 withstands Endo H treatment and is secreted as a heterogeneous complex-type *N*-glycosylated protein which is borderline-visible as an Endo H-resistant smear extending upward from around 50 kDa (Figure 2c). The higher sensitivity of deglycosylated IL-22BPi1 to detection in immunoblot also revealed the presence of secreted protein in the APs of untreated cells. This showed that both in the absence and presence of geldanamycin, IL-22BPi1 is secreted as a mixture of high-mannose and complex-type glycoforms, with both glycoform types increased in the presence of GA. IL-22BPi2, secreted at much higher levels than IL-22BPi1, was largely resistant to Endo H digestion, confirming its secretion as complex-type glycoprotein. In a separate experiment, we compared the secreted fraction of the three isoforms treated with PNGase F in the presence or absence of GA. Secretion of neither IL-22BPi3 nor IL-22BPi2 increased under GA treatment, in contrast to IL-22BPi1 (Figure 2d).

### 2.3. CsA Increases IL-22BPi1 Secretion

**Figure 3:**
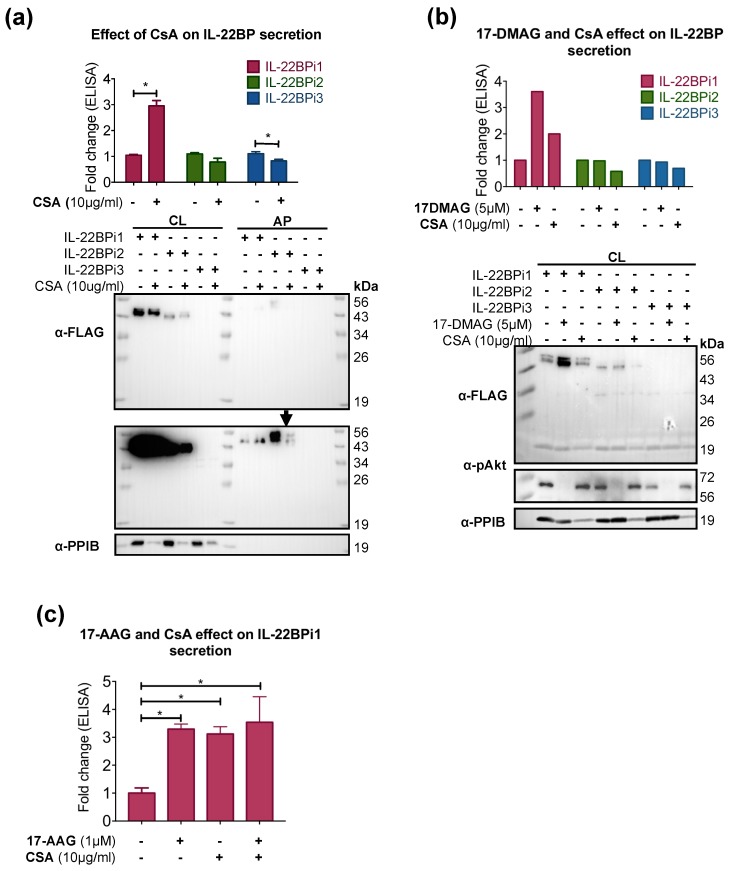
Cyclosporin A (CsA) enhances IL-22BPi1 secretion. (**a**) HEK293 cells were transiently transfected with the indicated expression plasmids and, 24 h later, cells were pretreated with cyclosporin A (CsA; 10 μg/mL) for 4 h or left untreated, medium was removed and cell monolayers washed five times with complete culture medium, and new complete culture medium containing CsA was added, whereby cells were incubated for a further 12 h, and the CM collected. Secreted IL-22BP was measured by ELISA in the CM (mean ± SEM; *n* = 3; * *p* < 0.05 by paired *t*-test). Next, the cell monolayers were carefully washed five times with prewarmed SFM to remove serum components, and fresh SFM containing L-glutamine was added for further 4 h prior to acetone precipitation, at which point the cells were lysed. Cell lysates (CL) and acetone precipitates (AP) were immunoblotted for FLAG and cyclophilin B (PPIB). IL-22BPi3 was detected in ELISA but not in Western blots, as previously shown [16]. (**b**) Shown is a representative experiment comparing the effect of 17-dimethylaminoethylamino-17-demethoxygeldanamyin (17-DMAG) and CsA on the intracellular and secreted levels of IL-22BP isoforms. HEK293 cells were transiently transfected with the indicated expression plasmids and, 24 h later, cells were pretreated, or not, with CsA (10 μg/mL) or 17-DMAG (5 μM) for 4 h, then medium was removed, cells were washed five times, new complete culture medium containing CsA or 17-DMAG was added, and cells were incubated for a further 12 h. CM was collected and cells were lysed. Secreted IL-22BP was measured by ELISA in the CM. CL were immunoblotted for FLAG, pAkt, and PPIB. 17-DMAG blocks Akt phosphorylation, validating its biological activity. (**c**) Effect of co-treatment with 1 μM 17-allylamino-17-demethoxygeldanamycin (17-AAG) and 10 μg/mL CsA on secretion of IL-22BPi1, as measured by ELISA. Cells were treated as in (a); mean ± SEM; *n* = 4; * *p* < 0.005 by paired *t*-test.

We evaluated the effect of CsA on the secretion of IL-22BP isoforms by transiently transfected HEK293 cells. CsA caused depletion of intracellular cyclophilin B by inducing its secretion, as previously reported [30,37]. CsA did not appreciably alter the intracellular levels of IL-22BPi1, and those of IL-22BPi2 were also unaffected (Figure 3a). However, while secretion of IL-22BPi2 and IL-22BPi3 appeared to be unaffected or suppressed, IL-22BPi1 secretion was significantly increased (Figure 3a). We also compared the effect of CsA with that of 17-DMAG on the comparative secretion of the three IL-22BP isoforms as measured by ELISA. 17-DMAG and CsA increased the secreted fraction of IL-22BPi1; however, CsA decreased the secretion of IL-22BPi2 and IL-22BPi3 while 17-DMAG did not seem not to exert any effect (Figure 3b). Used in combination, CsA and 17-AAG also increased the secretion of IL-22BPi1, however, this increase was not higher than that induced by the individual treatments (Figure 3c).

## 3. Discussion

The human *IL22RA2* gene co-expresses three transcript variants in immature moDCs, coding for three partially distinct proteins [15,16,31]. Of these, IL-22BPi2 and IL-22BPi3 are efficiently secreted and capable of neutralizing the biological activity of IL-22. IL-22BPi1, in contrast, does not bind to IL-22, is poorly secreted, retained in the ER, partially degraded by the proteasome, exhibits hallmarks of a misfolded protein, and is capable of inducing the UPR [16]. Interactome analysis revealed that both IL-22BPi1 and IL-22BPi2 are found in a complex with a series of ER chaperones, including GRP78, GRP94, calnexin, GRP170, PDIA6, cyclophilin B, and ERdj3 [16]. Structure–function and ensuing analyses showed that secretion of both IL-22BPi1 and IL-22BPi2, but not IL-22BPi3, is dependent on functionally intact GRP78 and GRP94 [16], and that IL-22BPi1 co-immunoprecipitates higher quantities of GRP94 than IL-22BPi2. In this article, we analyzed whether pharmacological targeting of GRP94 or cyclophilin B by GA analogs or CsA, respectively, can be translationally applied to modulate secretion of IL-22BP isoforms.

**Figure 4:**
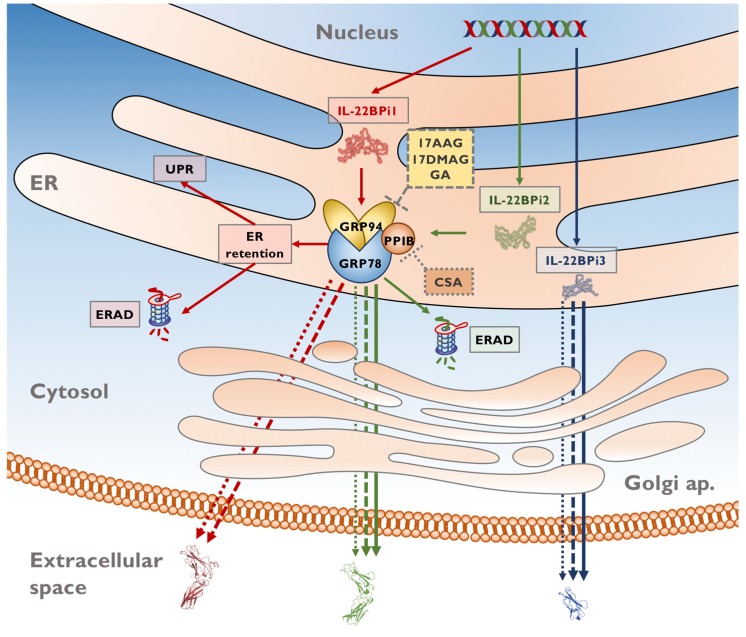
Pathways governing secretion and/or ER-associated degradation (ERAD) of IL-22BP isoforms. The diagram depicts the fates of IL-22BPi1, IL-22BPi2, and IL-22BPi3 in untreated transfected cells and following pharmacological targeting of the ER-resident chaperones GRP94 or cyclophilin B. IL-22BPi1 (red symbols and arrows), IL-22BPi2 (green symbols and arrows), and IL-22BPi3 (blue symbols and arrows) are produced through alternative splicing of primary *IL22RA2* transcripts. The three isoforms are directed to the ER through their shared signal peptide. IL-22BPi1 and IL-22BPi2 interact in the ER with GRP78, GRP94, and cyclophilin B (PPIB), among other ER-resident chaperones and foldases [16], while IL-22BPi3 appears not to exhibit these interactions. IL-22BPi1 is retained in the ER, delivered for degradation by the proteasome, and is capable of activating the unfolded protein response (UPR). IL-22BPi2 and IL-22BPi3 are more efficiently secreted; however, part of IL-22BPi2 was also found to be subject to proteasomal degradation. Cyclosporin A (CsA) causes depletion of intracellular cyclophilin B (dotted grey line) and geldanamycin (GA), and its analogs 17-dimethylaminoethylamino-17-demethoxygeldanamyin (17-DMAG) and 17-allylamino-17-demethoxygeldanamycin (17-AAG) bind to GRP94 in the ATP-binding site, inhibiting its ATPase activity (dashed grey line). In the presence of CsA or GRP94 inhibitors, IL-22BPi1 is mobilized to be secreted into the extracellular space. GA and analogs do not alter IL-22BPi2 and IL-22BPi3 secretion, but CsA decreases the secretion of both.

Interestingly, intracellular as well as secreted levels of IL-22BPi1 were strongly increased by GA or its analogs. Secretion of IL-22BPi1 was also enhanced by CsA. CsA does not seem to bind to IL-22BP in a bioinformatic study in which more than 100 putative CsA partners were identified [38]. IL-22BPi2 and IL-22BPi3 were not affected by GA or its analogs, while their secretion appeared to decrease in the presence of CsA (overview in Figure 4). Our results suggest that the source of extra secreted IL-22BPi1 protein is to be found in the fraction of IL-22BPi1 retained intracellularly and likely destined for degradation by the proteasome. This, in turn, indicates that, based on the results of this study, both intact GRP94 and cyclophilin B are instrumental in ER retention and ER-associated degradation (ERAD) of IL-22BPi1, and that unperturbed ATPase activity of GRP94—despite the demonstrated need for intact GRP94 for the secretion of both IL-22BPi1 and IL-22BPi2 [16]—is ultimately less crucial in facilitating the secretion of both isoforms than it is for ERAD of IL-22BPi1. Pertinent to this finding, the extra exon in IL-22BPi1 is a strong determinant of the interaction with GRP94; when cloned into the reading frame of the unrelated GRP94-non-binding cytokine IL-2, the resulting protein fails to be secreted and gains strong reactivity for GRP94 [16].

The existence of multimeric complexes in which cyclophilin B associates with other ER-resident proteins, such as GRP94, GRP78, and calreticulin, has been described [39,40,41]. The enzymatic activity of cyclophilin B has been shown to play crucial roles in ERAD and ER redox homeostasis [34,42,43]. GRP94 is also thought to be an essential player in ERAD [25,44,45]. In fact, GRP94 displays a preference for interacting with non-secretable proteins [46]. Several studies have documented GRP94′s capacity to bind to folding intermediates so as to prevent them from degradation. GA treatment reduces both intracellular and secreted amounts of bile salt-dependent lipase (BSDL), suggesting that dissociation of BSDL from GRP94 reverts this protein from the secretory to the degradation pathway [47], and similar observations were made for another client, p185-erB2 [48]. By contrast, IL-22BPi1 joins mutant a1-antitrypsin [44] and γ-aminobutyric acid type A receptors [45] in revealing GRP94 as a crucial factor for sequestering misfolded proteins in the ER, positively regulating their ERAD.

Apart from IL-22BPi1, IL-22BPi2 but not IL-22BPi3 was found to be partially targeted for degradation by the proteasome [16]. However, while IL-22BPi2 is efficiently secreted, isoform 1 is not, suggesting that the rate of ERAD for the long isoform must be much higher than for the canonical isoform. The effect of GA analogs, together with that of CsA, underscores that an orchestrated ERAD response comprising different ER-resident chaperones is needed for IL-22BPi1 degradation, and that perturbance of this multimeric chaperone complex by pharmacologically targeting its specific components leads isoform 1 to “reroute” from the ERAD to the secretory pathway. Deglycosylation analysis showed that IL-22BPi1 is secreted as a mixture of high-mannose and complex-type glycoforms, which validates its use in the classical secretory route. Decreased secretion of IL-22BPi2 and IL-22BPi3 in the presence of CsA may be related to changes in ER oxidative pathways [34] or peptidylprolyl isomerase activity.

In conclusion, PPIB and GRP94 ATPase activity may be co-involved in ER sequestration and ERAD of IL-22BPi1, with pharmacological impairment of these enzymatic activities by GA analogs or CsA inducing secretion of IL-22BPi1.

## 4. Materials and Methods

### 4.1. Monocyte Isolation and Monocyte-Derived Dendritic Cell Differentiation

Fresh peripheral blood mononuclear cells (PBMCs) were obtained from buffy coats provided by the Basque biobank (www.biobancovasco.org/en/) with the approval of the ethics committee of clinical investigation of the Basque Country (CEIC-E; CEIC-E-3000911, 28 September 2017). PBMCs were isolated by Ficoll-Paque (GE Healthcare, Pittsburgh, PA, USA) density centrifugation. CD14^+^ monocytes were purified by positive selection with MACS CD14 microbeads (130-050-201, Miltenyi, Bergisch Gladbach, Germany) following manufacturer’s instruction. Monocytes were cultured in Mo-DC differentiation medium (130-094-812, Miltenyi) at a density of 1 × 10^6^ cells/mL for three days; at this point, an equal volume of fresh medium was added and cells were incubated until day 6 at 37 °C in a humidified incubator supplying 5% CO_2_/air. Lipopolysaccharide (LPS; L6143, Sigma, St. Louis, MO, USA; 200 ng/mL) addition was performed on day 6, and cells left for 12 or 24 h. Cells were stained with Mo-DC Differentiation Inspector (130-093-567, Miltenyi) and analyzed with MacsQuant flow cytometer following manufacturer’s instructions.

### 4.2. RNA Extraction and qPCR

RNA extraction was performed with TRI Reagent (Sigma, T9424) following manufacturer’s instruction and reverse transcribed using the High-Capacity cDNA Reverse Transcription Kit (4368814, ThermoFisher Scientific, Waltham, MA, USA), following the manufacturer’s instructions and using a Veriti thermocycler (Applied Biosystems, Waltham, MA, USA). Quantitative PCR (qPCR) was performed using a 7500 Fast Real Time PCR System (Applied Biosystems). The primers used in this study are the following: IL22RA2 (Hs00364814_m1; FAM) and ACTB (hS99999903_m1; VIC), all purchased from ThermoFisher Scientific).

### 4.3. Cell Culture and Transfection

HEK293 cells were cultured in Dulbecco’s Modified Eagle Medium (DMEM; D5796, Sigma) supplemented with 10% fetal bovine serum (FBS; F9665, Sigma), which corresponds to the complete culture medium referred to in the text. Cells were incubated at 37 °C in a humidified incubator supplying 5% CO_2_/air. Cells were plated on a 24-well plate at 500 µL/well at density of 3 × 10^4^ cells/well in the indicated growth medium and propagated to 60%–70% confluency at the time of transfection using MACSfectin Reagent (130-098-412, Miltenyi). *IL22RA2i2* and *IL22RA2i3* expression plasmids were purchased from OriGene (RC219095, Rockville, MD, USA) and GenScript (Ohu00490, Piscataway, NJ, USA), respectively. *IL22RA2i1* was constructed as described in our previous study [16].

### 4.4. Drug Treatment

All drugs were added to the cell cultures at 24 h after transfection. Cyclosporine A (CsA; 30024, Sigma) was added at 10 μg/mL and 17-allylamino-17-demethoxygeldanamycin (17-AAG), 17-dimethylaminoethylamino-17-demethoxygeldanamyin (17-DMAG), and geldanamycin (GA) (ant-agl-5, ant-dgl-5 and ant-gl, Invivogen, San Diego, CA, USA) were added at either 1 or 5 μM in complete culture medium for 4 h, then medium was removed and cells were carefully washed five times with complete culture medium to remove any remaining secreted IL-22BP isoforms produced prior to drug treatment; new complete culture medium containing CsA, 17-AAG, 17-DMAG, or GA was added and cells were incubated for the indicated times.

### 4.5. Protein Extraction, Deglycosylation, Immunoblotting, and ELISA

Cells and CM were collected at the indicated times. For acetone precipitation of conditioned media, cells were treated with the indicated compounds for 16 h, CM was removed, cells were carefully washed five times with prewarmed serum-free medium (SFM; 12-764Q, Lonza, Basel, Switzerland) to remove abundant serum proteins, and fresh SFM containing L-glutamine (G5792, Sigma) was added for a further 4 h prior to acetone precipitation and harvest of cells for lysis. Conditioned media were acetone-precipitated with four volumes of ice-cold acetone and incubated on ice for 10 min followed by centrifugation at 21,000 *g* at 4 °C. Pellets were resuspended in deglycosylation or SDS-PAGE reducing loading buffer. Deglycosylation enzymes, EndoH and PNGaseF, were purchased from New England Biolabs (P0702 and P0704, Ipswich, MA, USA) and used following manufacturer’s instructions. Cells were washed three times with cold PBS and lysed in RIPA buffer (25 mM Tris-HCl, 150 mM NaCl, pH 7.6; plus 1% NP-40, 1% sodium deoxycholate, and 0.1% SDS) in presence of protease inhibitors (11697498001, Roche, Basel, Switzerland) for 30 min on ice. Cells were centrifuged at 21,000 *g* at 4 °C for 10 min. Supernatants were collected and total protein was quantified using a BCA kit (23225, ThermoFisher Scientific) andfollowing manufacturer’s instructions. Equal amounts of total protein from cell lysates (CL) and equal volumes of conditioned precipitated media were loaded in reducing conditions on SDS-PAGE gels (10% acrylamide) and resolved, transferred to PVDF membranes (IPVH00010, Millipore, Burlington, MA, USA), and immunoblotted. For immunoblotting, the antibodies used in this study are the following: anti-FLAG (1:1000, 2043-1-AP, Proteintech, Rosemont, IL, USA); anti-IL-22BP (1:1000, AF1087, R&D Systems, Minneapolis, MN, USA); anti-GRP94 (1:1000, ADI-SPA-850, Enzo, Farmingdale, NY, USA); anti-pAkt (1:500, 9271, Cell signaling, Danvers, MA, USA); anti-PPIB (1:5000, 16045, Abcam, Cambridge, UK); anti-actin (1:1000, A2066, Sigma); anti-PPIC (1:1000, 10287-2-AP, Proteintech); anti-tubulin (1:1000, A01490, GenScript); and all HRP-conjugated secondary antibodies were purchased from Jackson ImmunoResearch. ELISA was performed as previously described in [16].

### 4.6. Statistical Analysis

Data presented are means ± SEM. Statistical significance was taken as *p* < 0.05. Data analysis was carried out by performing *t*-tests using GraphPad Prism version 7, (GraphPad Software, La Jolla, CA, USA).

## Abbreviations

17-AAG	17-allylamino-17-demethoxygeldanamycin
17-DMAG	17-dimethylaminoethylamino-17-demethoxygeldanamycin
AP	acetone precipitates
CL	cell lysates
CM	conditioned medium
CsA	cyclosporin A
DM	differentiation medium
Endo H	endonuclease H
ER	endoplasmic reticulum
ERAD	ER-associated degradation
GA	geldanamycin
GRP94	glucose-regulated protein 94
GRP78	glucose-regulated protein 78
IL-22BP	interleukin-22 binding protein
IL22RA2	interleukin-22 receptor alpha-chain 2
moDC	monocyte-derived dendritic cell
PPIB	peptidylprolyl isomerase B (cyclophilin B)
PPIC	peptidylprolyl isomerase C (cyclophilin C)
SFM	serum-free medium
UPR	unfolded protein response
